# Aberrantly expressed HORMAD1 disrupts nuclear localization of MCM8–MCM9 complex and compromises DNA mismatch repair in cancer cells

**DOI:** 10.1038/s41419-020-2736-1

**Published:** 2020-07-09

**Authors:** Kang Liu, Yifan Wang, Quanfeng Zhu, Peng Li, Jiyuan Chen, Zhenghui Tang, Yuanming Shen, Xiaodong Cheng, Lin-Yu Lu, Yidan Liu

**Affiliations:** 1 Key Laboratory of Reproductive Genetics (Ministry of Education) and Women’s Reproductive Health Laboratory of Zhejiang Province, Women’s Hospital, Zhejiang University School of Medicine, Hangzhou, China; 2 Institute of Translational Medicine, Zhejiang University School of Medicine, Hangzhou, China; 3 Department of Gynecologic Oncology, Women’s Hospital, Zhejiang University School of Medicine, Hangzhou, China

**Keywords:** Cancer genetics, DNA mismatch repair

## Abstract

HORMAD1 is a meiosis-specific protein that promotes synapsis and recombination of homologous chromosomes in meiotic prophase. Originally identified as a cancer/testis antigen, HORMAD1 is also aberrantly expressed in several cancers. However, the functions of HORMAD1 in cancer cells are still not clear. Here, we show that HORMAD1 is aberrantly expressed in a wide variety of cancers and compromises DNA mismatch repair in cancer cells. Mechanistically, HORMAD1 interacts with MCM8–MCM9 complex and prevents its efficient nuclear localization. As a consequence, HORMAD1-expressing cancer cells have reduced MLH1 chromatin binding and DNA mismatch repair defects. Consistently, HORMAD1 expression is associated with increased mutation load and genomic instability in many cancers. Taken together, our study provides mechanistic insights into HORMAD1’s functions in cancer cells, which can potentially be exploited for targeted therapy of HORMAD1-expressing cancers.

## Introduction

HORMAD1 is a meiosis-specific protein. In meiotic prophase, HORMAD1 promotes the formation of synaptonemal complex and facilitates the synapsis and recombination between homologous chromosomes^1–4^. Consistent with its important roles in meiosis, both male and female *Hormad1* knockout (KO) mice are infertile^3–5^.

Although the physiological functions of HORMAD1 are restricted to meiosis, HORMAD1 was originally identified as a cancer/testis antigen (CT46)^6^. Cancer/testis antigens are a group of proteins that are specifically expressed in testis but are aberrantly expressed in cancers. Later studies have confirmed that HORMAD1 is aberrantly expressed in several cancers, including gastric cancers^7^, lung cancers^8,9^, basal type and triple-negative breast cancers^10–14^, and ovarian cancers^15^. Aberrant HORMAD1 expression is caused by promoter hypomethylation^8,13,14^, which is believed to be unselected due to genome-wide loss of DNA methylation in many cancers^16^.

Studies have revealed that HORMAD1 can actively participate in cellular activities in cancers. Triple-negative breast cancers with aberrant HORMAD1 expression have frequent allelic-imbalanced copy-number aberrations (AiCNA), suggesting that HORMAD1 expression is associated with genomic instability^12^. Studies from the same group have found that HORMAD1-expressing cancer cells have decreased the efficiency of homologous recombination (HR) repair and increased sensitivity to cisplatin and PARP inhibitors (PARPi)^12^, suggesting that aberrantly expressed HORMAD1 compromises HR and promotes response to chemotherapy. However, opposite observations have been obtained from three recent studies. Ectopic HORMAD1 expression in some basal-like breast cancer cells decreases the sensitivity of these cells to PARPi in xenograft models^14^. In lung adenocarcinoma cells with aberrant HORMAD1 expression, HORMAD1 depletion causes HR deficiency and increased sensitivity to ionizing radiation or PARPi^8,9^. These three studies suggest that aberrantly expressed HORMAD1 promotes HR and chemoresistance.

Inconsistent results from the above studies suggest that the function of HORMAD1 in cancer cells remains elusive and requires further investigation. In this study, we report that aberrantly expressed HORMAD1 interacts with MCM8–MCM9 complex in cancer cells. We further reveal that HORMAD1 compromises DNA mismatch repair by preventing efficient nuclear localization of MCM8–MCM9 complex and reducing chromatin binding of MLH1, the key component of DNA mismatch repair machinery.

## Results

### HORMAD1 is widely expressed in cancers

**Fig. 1:**
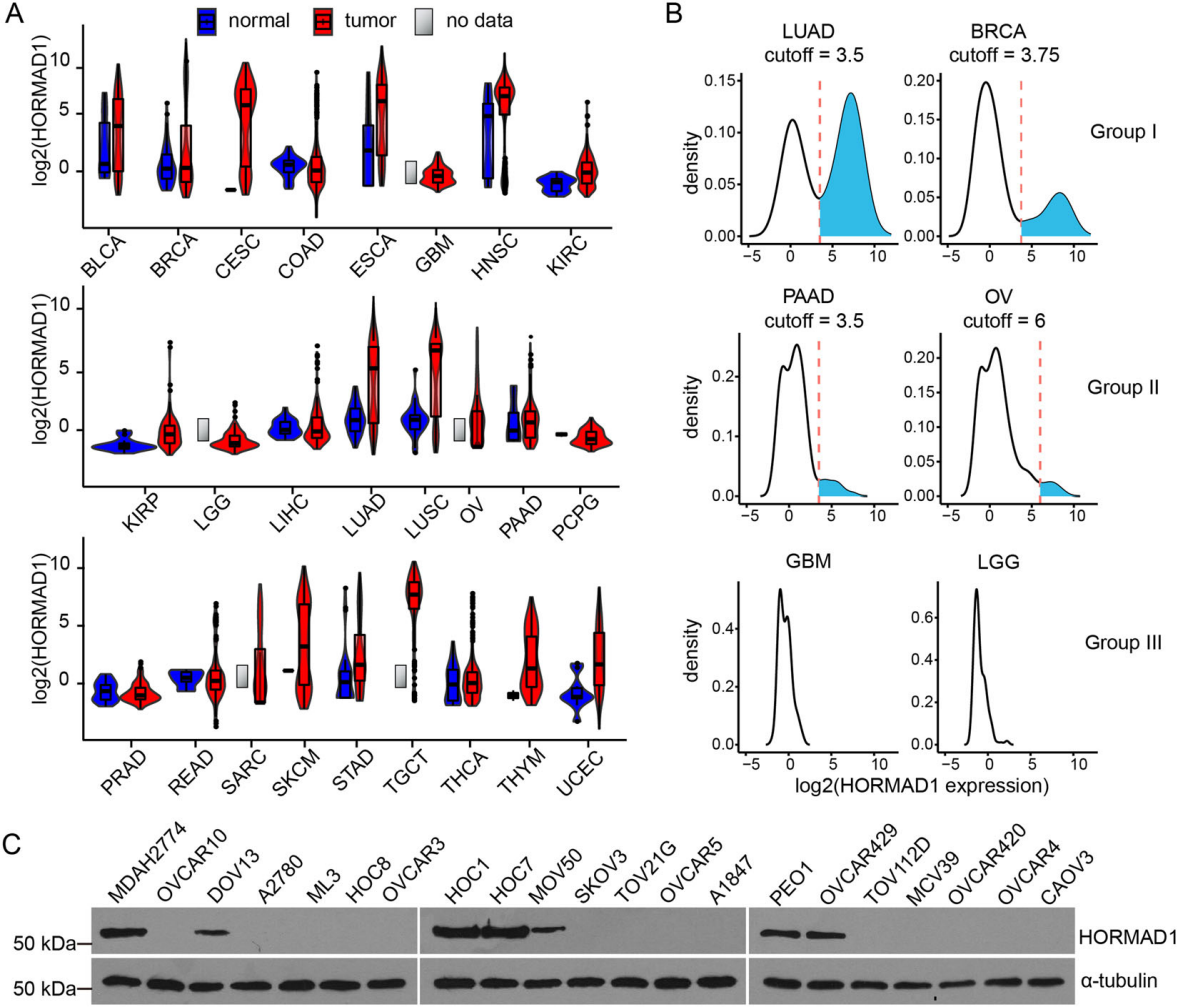
HORMAD1 is widely expressed in cancers. **a** Violin and box plots of log2-transformed HORMAD1 expression in TCGA cancers (red) with their normal samples (blue) as control. Cancers with more than 50 samples (*n* > 50) were analyzed. **b** Log2-transformed HORMAD1 expression distribution in typical examples of cancers from three groups of cancer types. Red line represents for the cutoff between HORMAD1-positive (expressed) and HOMRAD1-negative (silent) samples and the HORMAD1-positive area was filled blue. **c** Western blotting analyses of HORMAD1 expression in ovarian cancer cell lines.

Originally identified as a cancer/testis antigen, HORMAD1 is aberrantly expressed in several cancers. In order to examine the expression of HORMAD1 in cancers thoroughly, we conducted pan-cancer analysis of HORMAD1 expression in 25 types of cancers using RNA sequencing data from The Cancer Genome Atlas (TCGA) (Fig. 1a). In physiological conditions, HORMAD1 expression is restricted to meiotic cells in testes and ovaries. Indeed, HORMAD1 expression was low in most normal samples of different tissue origins, but was high in most samples in testicular germ cell tumors (TCGT) (Fig. 1a). Analyses of the rest 24 cancer types revealed that HOMRAD1 high expression could be found in most cancer types (Fig. 1a), suggesting that HORMAD1 is widely expressed in cancers.

To examine the expression patterns of HORMAD1 in cancers, density plot was applied to each cancer. Twenty-four types of cancers could be divided into three sub-groups based on the distribution of HOMRAD1 expression (Figs. 1b, S1). In group I, HORMAD1 expression plot showed an approximately bimodal shape (Figs. 1b, S1), suggesting that each type of cancer in this group contains two distinct populations: HORMAD1-positive and HORMAD1-negative cancers. In group II, HORMAD1 distribution plot did not show an apparent second peak, but the distribution was right-skewed with a small but significant percentage of cancer samples having high HORMAD1 expression (percentage > 4.5%) (Figs. 1b, S1). In group III, HORMAD1 expression was normally distributed without any outliers having high expression (Figs. 1b, S1).

Twelve types of cancers were in group I, including lung adenocarcinoma (LUAD) and breast invasive carcinoma (BRCA), in which HORMAD1 high expression has been confirmed by previous studies^8–14^. Six types of cancers were in group II including ovarian serous cystadenocarcinoma (OV). Since only 4.85% of the OV samples (15 out of 309 samples) had distinct high levels of HORMAD1 expression, we further examined HOMRAD1 expression in ovarian cancer cell lines. 7 out of 21 (30%) ovarian cancer cell lines analyzed had strong HORMAD1 expression (Fig. 1c), suggesting that HORMAD1 is indeed highly expressed in some ovarian cancers and the percentage of HORMAD1-expressing ovarian cancers might be underestimated. Therefore, a small but non-negligible percentage of samples within each type of group II cancers has high expression of HORMAD1. Taken together, HORMAD1 is widely expressed in group I and II cancers that include 18 cancer types and a large number of cancer samples.

### HORMAD1 interacts with MCM8–MCM9 complex

**Fig. 2:**
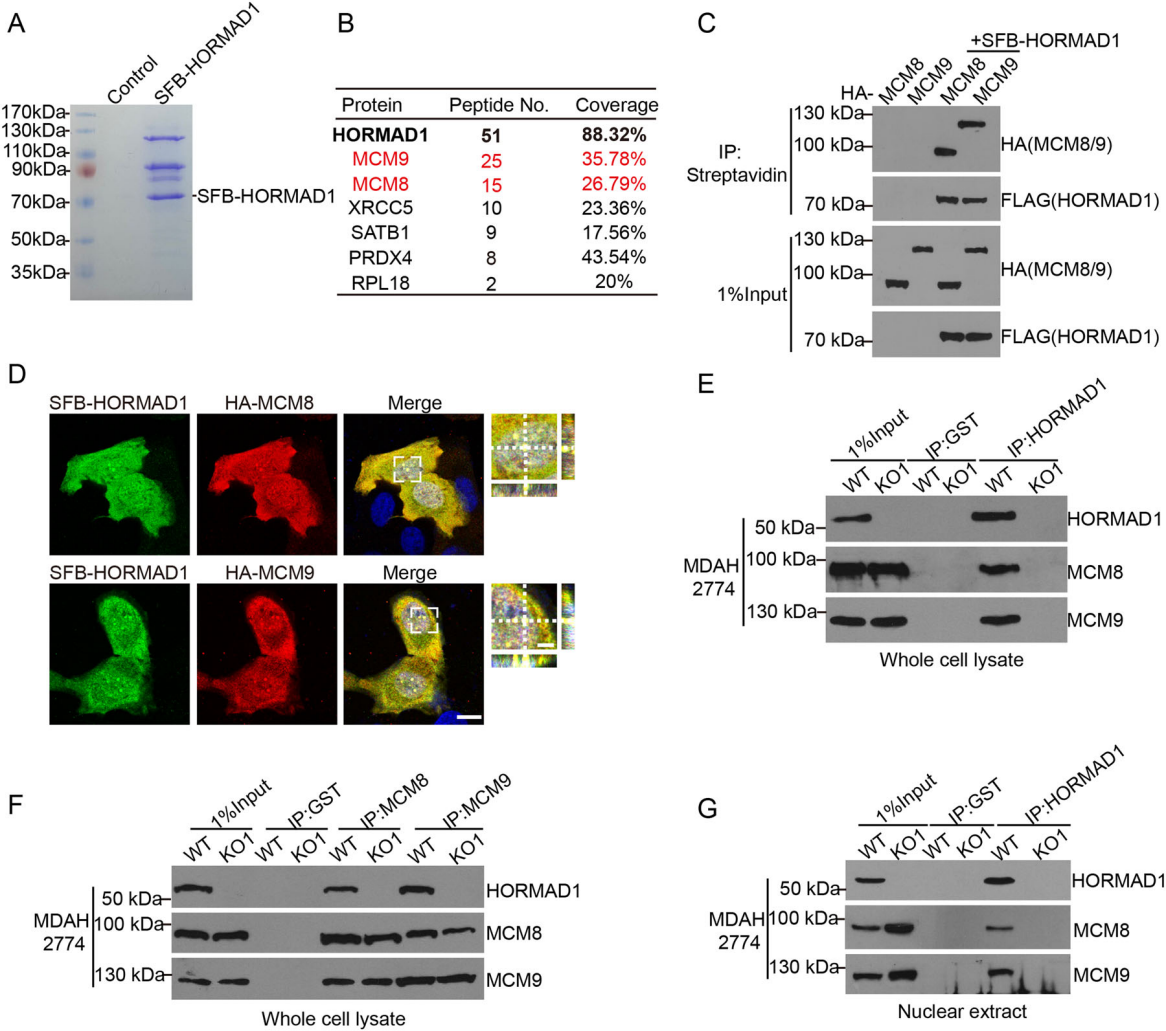
HORMAD1 interacts with MCM8-MCM9 complex. **a** 293T cells stably expressing either the vector control or SFB-tagged HORMAD1 were used for tandem affinity purification. The final eluates were separated by SDS-PAGE gels and stained with Coomassie blue. **b** A list of HORMAD1 interacting proteins identified by mass spectrometry is shown. The complete list is shown in Table S1. **c** Co-IP analyses of SFB-tagged HORMAD1 and HA-tagged MCM8 or MCM9 in 293T cells. **d** Confocal microscopy analyses of SFB-tagged HORMAD1 and HA-tagged MCM8 or MCM9 in U2OS cells. The cut view panel depicts two perpendicular transverse sections as indicated by white lines, intersecting at the point of the brightest fluorescence signal. Scale bars are 20 μm (left) and 5 μm (right), respectively. **e**–**g**. Co-IP analyses of endogenous HORMAD1 and MCM8–MCM9 complex using indicated antibodies in WT and HORMAD1 KO MDAH2774 cells in whole cell lysate (**e**, **f**) and nuclear extract (**g**).

To provide mechanistic insights into the function of HORMAD1 in cancer cells, we established 293T cells stably expressing S-Flag-streptavidin binding protein (SFB)-tagged HORMAD1 and performed tandem affinity purification and mass spectrometry analysis. MCM8 and MCM9, which form a stable complex, were among the most abundant proteins identified (Fig. 2a, b, Table S1), suggesting that HORMAD1 might have a functional link with MCM8–MCM9 complex in cancer cells.

To verify the results of tandem affinity purification, we first examined the interaction using exogenously tagged proteins. SFB-tagged HORMAD1 interacted with both HA-tagged MCM8 and MCM9 (Fig. 2c). Confocal microscopy analysis also revealed that SFB-tagged HORMAD1 colocalized with both HA-tagged MCM8 and MCM9 in cells (Fig. 2d). Reciprocal co-immunoprecipitation (co-IP) experiments further confirmed the interaction between endogenous HORMAD1 and MCM8–MCM9 complex in HORMAD1-positive MDAH2774 cells in both whole cell lysates and nuclear extract (Fig. 2e–g). Importantly, the interaction could not be detected in HORMAD1 KO MDAH2774 cells (Fig. 2e–g), suggesting that the endogenous interaction was specific. Collectively, these data strongly support that MCM8–MCM9 complex is a binding partner of HORMAD1 in cancer cells.

### HORMAD1’s HORMA domain binds a HORMAD1-interacting motif (HIM) at the C-terminus of MCM9

**Fig. 3:**
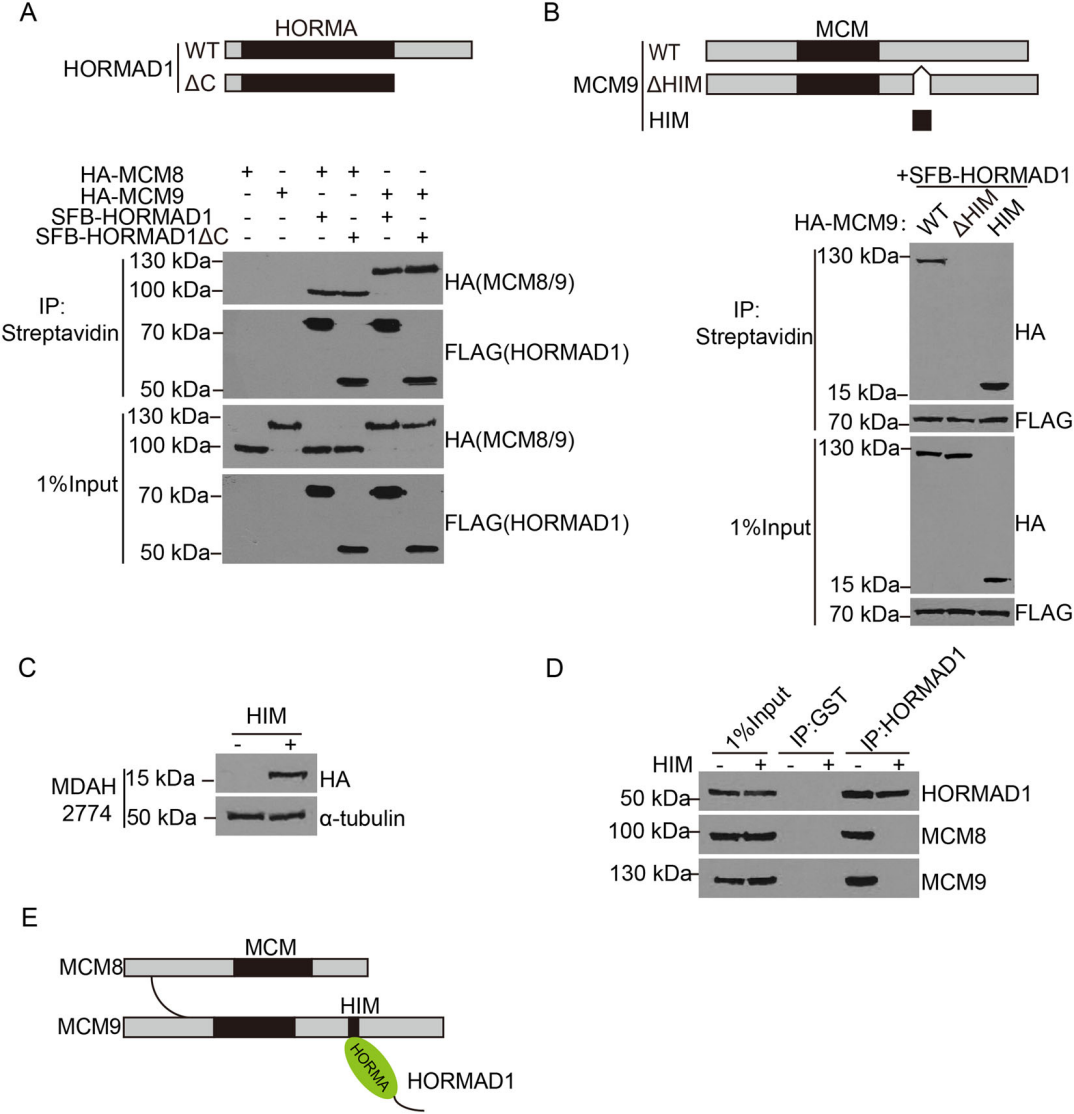
HORMAD1’s HORMA domain binds a HORMAD1-interacting motif (HIM) at the C-terminus of MCM9. **a** Upper, A schematic representation of WT and deletion mutants of HORMAD1. Lower, Co-IP analyses of SFB-tagged HORMAD1 deletion mutants and HA-tagged MCM8 or MCM9 in 293T cells. **b** A schematic representation of MCM9 WT, ΔHIM, and HIM (HORMAD1 interacting motif) is shown. Co-IP analyses of the interaction between SFB-HORMAD1 and HORMAD1 HIM of MCM9 in 293T cells. **c** Western blotting analyses of HA in MDAH2774 cells with or without HA-MCM9 HIM. *α*-tubulin was used as loading control. **d** Co-IP analyses of endogenous HORMAD1 and MCM8–MCM9 complex in MDAH2774 cells with or without HA-MCM9 HIM. **e** A model of the interaction between HORMAD1 and MCM8–MCM9 complex.

To examine how HORMAD1 binds MCM8–MCM9 complex, we further mapped the domain required on each protein for the interaction. Removing the C-terminal intrinsically disordered region of HORMAD1 did not affect MCM8–MCM9 complex binding, suggesting that the interaction was mediated by the N-terminal HORMA domain (Fig. 3a). A series of domain mapping experiments revealed that MCM8 and MCM9 dimerized through their N-terminal regions (Fig. S2A–D), and HORMAD1 interacted with MCM8–MCM9 complex through a small region at the C-terminus of MCM9 (amino acids 703–766, referred to as HORMAD1-interacting motif (HIM) hereinafter) (Fig. S2E–H). Indeed, MCM9 HIM alone was sufficient for interacting with HORMAD1 (Fig. 3b). Importantly, when MCM9 HIM was stably expressed in MDAH2774 cells (Fig. 3c), the interaction between endogenous HORMAD1 and MCM8–MCM9 complex could not be detected (Fig. 3d). It is likely that MCM9 HIM overexpression saturates HORMAD1 binding and releases endogenous MCM8–MCM9 complex. Therefore, these domain mapping experiments reveal that HORMAD1 interacts with MCM8–MCM9 complex through the binding between its HORMA domain and MCM9 HIM (Fig. 3e).

### HORMAD1’s function in HR repair is independent of its interaction with MCM8–MCM9 complex

The interaction between HORMAD1 and MCM8–MCM9 complex suggest that the function of HORMAD1 in cancer cells might be linked with MCM8–MCM9 complex. MCM8–MCM9 complex functions in both HR repair and DNA mismatch repair^17–20^. Since previous studies have suggested that HORMAD1 might function in HR repair^8,9,12,14^, we first investigated if HORMAD1 and MCM8–MCM9 complex have functional links in HR repair. Due to inconsistency between results obtained from different groups in previous studies^8,9,12,14^, we performed an independent evaluation HORMAD1’s function in HR repair in cancer cell lines using a DR-GFP plasmid reporter (Fig. S3A). HORMAD1 expression increased HR efficiency in HORMAD1-negative ovarian cancer cell line OVCAR5, while HORMAD1 KO decreased HR efficiency in HORMAD1-expressing ovarian cancer cell line MDAH2774 (Fig. S3B–C, E–F). PARP inhibitor (PARPi) sensitivity is another indicator of HR status. Consistently, HORMAD1 expression decreased PARPi sensitivity in OVCAR5 cells (Fig. S3D), while HORMAD1 KO increased PARPi sensitivity in MDAH2774 cells (Fig. S3G). Similar results were obtained in non-small-cell lung cancer cell line A549 and triple-negative breast cancer cell line HCC38 that were used in previous studies (Fig. S3H–M). Together, consistent with most studies, these observations suggest that HORMAD1 promotes HR repair in cancer cells.

Since both HORMAD1 and MCM8–MCM9 complex promotes HR, we continued to examine if HORMAD1 promotes HR through its interaction with MCM8–MCM9 complex. However, when the interaction between endogenous HORMAD1 and MCM8–MCM9 complex was abolished in MDAH2774 cells stably expressing MCM9 HIM, the HR efficiency and PARPi sensitivity remained unaltered, which was different from the decreased HR and increased PARPi sensitivity in HORMAD1 KO MDAH2774 cells (Fig. S3N–O). Therefore, HORMAD1’s function in HR repair is independent of its interaction with MCM8–MCM9 complex.

We further examined the recruitment of key HR enzyme RAD51 to DNA break sites. Interestingly, HORMAD1 expression did not affect RAD51 foci in OVCAR5 cells (Fig. S4A). HORMAD1 KO did not affect RAD51 foci in MDAH2774, A549, or HCC38 cells either (Fig. S4B–D). It is likely that HORMAD1 promotes HR repair through ways other than regulating the recruitment of RAD51 to DNA break sites. The mechanism how HORMAD1 promotes HR repair in cancer cells requires further investigation in future.

### HORMAD1 compromises DNA mismatch repair through its interaction with MCM8–MCM9 complex

**Fig. 4:**
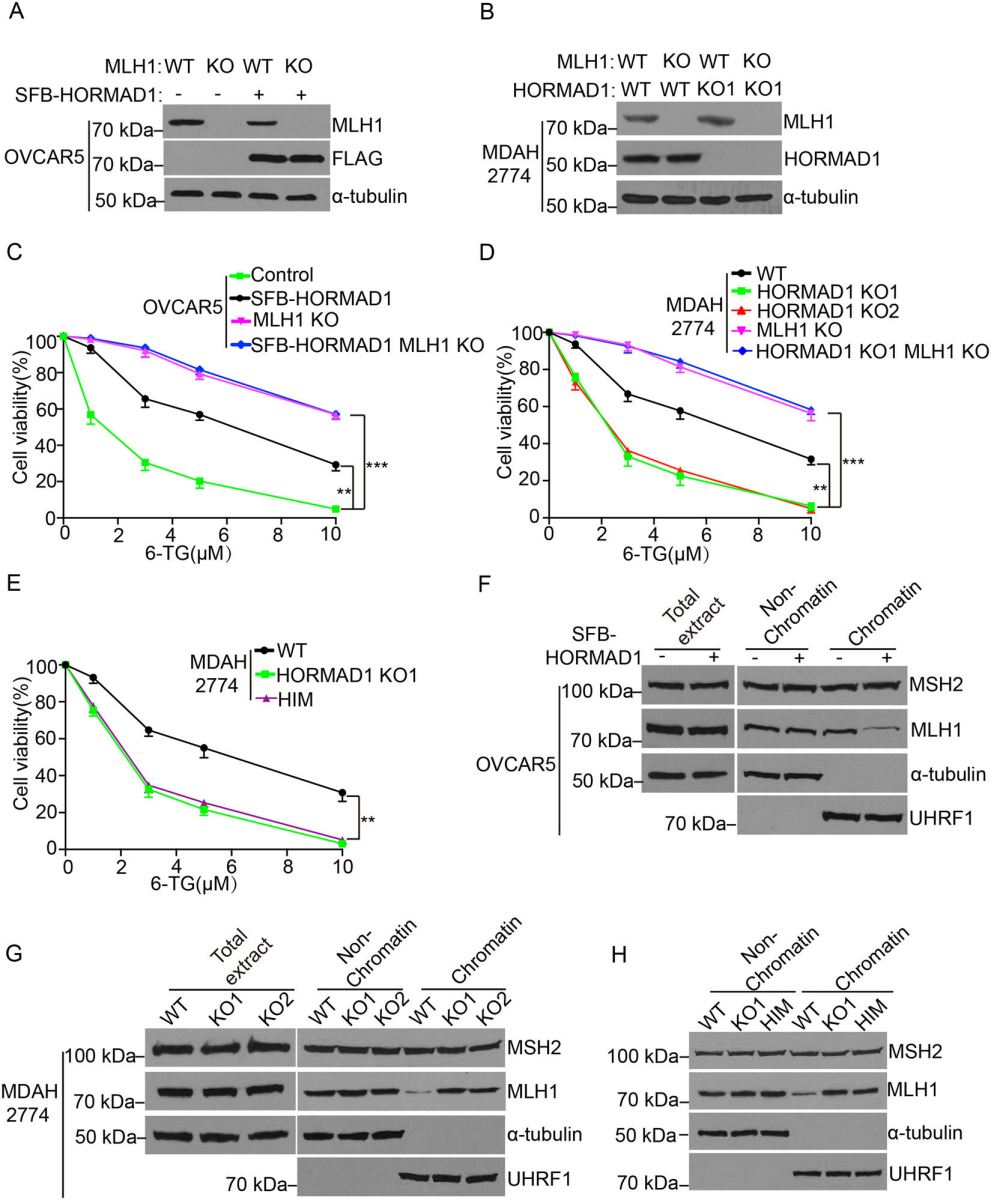
HORMAD1 compromises DNA mismatch repair. **a**, **b** Western blotting analyses of MLH1 proteins in OVCAR5 cells with or without HORMAD1 (**a**) and WT and HORMAD1 KO MDAH2774 cells (**b**). *α*-tubulin was used as loading control. **c**, **d**. Cell viability of OVCAR5 cells with or without HORMAD1 (**c**) and WT and HORMAD1 KO MDAH2774 cells (**d**) after treatment with indicated doses of 6-TG. MLH1 KO was used as control. **e** Cell viability analyses of MDAH2774 cells with or without HA-MCM9 HIM after treatment with indicated doses of 6-TG. **f** Western blotting analyses of total, chromatin-bound, and non-chromatin-bound MSH2 and MLH1 proteins in OVCAR5 cells with or without HORMAD1. *α*-tubulin and UHRF1 were used as loading controls for non-chromatin and chromatin fractions, respectively. **g** Western blotting analyses of total, chromatin-bound, and non-chromatin-bound MSH2 and MLH1 proteins in WT and HORMAD1 KO MDAH2774 cells. α-tubulin and UHRF1 were used as loading controls for nonchromatin and chromatin fractions, respectively. **h** Western blotting analyses of chromatin bound and nonchromatin bound MSH2 and MLH1 proteins in MDAH2774 cells with or without HA-MCM9 HIM. α-tubulin and UHRF1 were used as loading controls for non-chromatin and chromatin fractions, respectively. Mean ± SEM from three independent experiments are shown. **p < 0.01; ***p < 0.001.

Besides HR repair, MCM8–MCM9 complex is also important for DNA mismatch repair^20^. Therefore, we investigated if HORMAD1 and MCM8–MCM9 complex have functional links in DNA mismatch repair. We first employed the classic *HPRT* gene mutation assay to examine the influence of HORMAD1 expression on DNA mismatch repair efficiency. Cell with DNA mismatch repair deficiency are more resistant to 6-thioguanine (6-TG) due to elevated induced mutation frequencies in *HPRT* gene^21^. MLH1 KO were generated in OVCAR5 and MDAH2774 cells and were used in this assay as a positive control (Fig. 4a, b). Interestingly, HORMAD1 expression decreased 6-TG sensitivity in OVCAR5 cells (Fig. 4c), while HORMAD1 KO increased 6-TG sensitivity in MDAH2774 (Fig. 4d). Compared with HORMAD1-expressing cells, MLH1 KO cells have further decreased 6-TG sensitivity. Importantly, HORMAD1 expression did not further decreased 6-TG sensitivity of MLH1 KO OVCAR5 cells, and HORMAD1 KO failed to increased 6-TG sensitivity in MLH1 KO MDAH2774 cells (Fig. 4c, d). These results suggest that effect of HORMAD1 on DNA mismatch repair is synergistic to MLH1 KO. Similar results were obtained in A549 and HCC38 cells (Fig. S5A, B). Collectively, these results reveal that HORMAD1 expression compromises DNA mismatch repair in cancer cells, and the effect of HORMAD1 expression is roughly half of MLH1 KO.

When the interaction between endogenous HORMAD1 and MCM8–MCM9 complex was abolished in MDAH2774 cells stably expressing MCM9 HIM, the 6-TG sensitivity was significantly increased to a level similar to that in HORMAD1 KO MDAH2774 cells (Fig. 4e). This observation strongly suggests that HORMAD1 compromises DNA mismatch repair through its interaction with MCM8–MCM9 complex. To further interrogate this possibility, we examined how HORMAD1 compromise DNA mismatch repair pathway.

Human DNA mismatch repair machinery consists of MutS homologs (MSH2/3 and MSH2/MSH6 heterodimers) that recognize mismatches and MutL homologs (MLH1/PMS2, MLH1/PMS1, and MLH1/MLH3 heterodimers) that cleave the DNA strand containing mismatches^22^. MCM8–MCM9 complex is downstream of MSH2 and is important for MLH1 chromatin loading^20^. In the absence of MCM8–MCM9 complex, the chromatin loading of MLH1, but not MSH2, are significantly decreased^20^. The expression levels of MSH2 and MLH1 were not affected by HORMAD1 expression (Figs. 4f–g, S5C–D). Interestingly, chromatin binding of MLH1, but not MSH2, was significantly reduced in OVCAR5 cells after HORMAD1 expression (Fig. 4f). Consistently, HORMAD1 KO elevated chromatin binding of MLH1, but not MSH2, in MDAH2774, A549, and HCC38 cells (Figs. 4g, S5C–D). Therefore, HORMAD1 expression compromises DNA mismatch repair by interfering chromatin binding of MLH1 in cancer cells, which is similar to cells without MCM8–MCM9 complex. Importantly, when the interaction between endogenous HORMAD1 and MCM8–MCM9 complex was abolished in MDAH2774 cells stably expressing MCM9 HIM, the chromatin level of MLH1 was restored to a level similar to that in HORMAD1 KO MDAH2774 cells (Fig. 4h). These observations further demonstrate that HORMAD1 compromises DNA mismatch repair through its interaction with MCM8-MCM9 complex.

### HORMAD1 compromises nuclear localization of MCM8–MCM9 complex

**Fig. 5:**
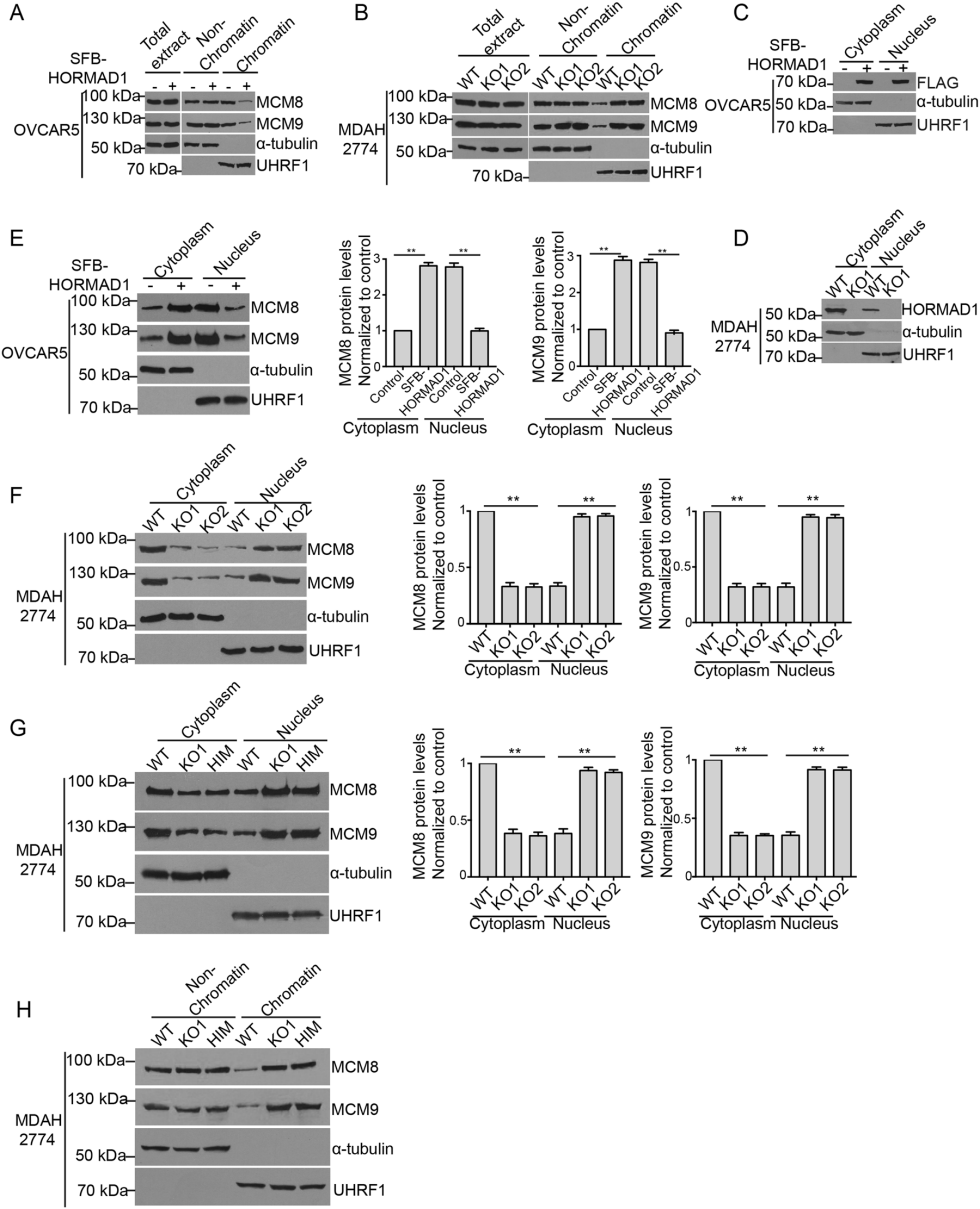
HORMAD1 compromises nuclear localization of MCM8–MCM9 complex that leads to mismatch repair defects. **a**, **b** Western blotting analyses of total, chromatin-bound, and nonchromatin-bound MCM8 and MCM9 proteins in OVCAR5 cells with or without HORMAD1 (**a**) and WT and HORMAD1 KO MDAH2774 cells (**b**). *α*-tubulin and UHRF1 were used as loading controls for non-chromatin and chromatin fractions, respectively. **c**, **d** Western blotting analyses of cytosolic and nuclear HORMAD1 protein in OVCAR5 cells with or without HORMAD1 (**c**) and WT and HORMAD1 KO MDAH2774 cells (**d**). *α*-tubulin and UHRF1 were used as loading controls for cytoplasm and nucleus, respectively. **e**, **f**. Western blotting analyses of cytosolic and nuclear MCM8 and MCM9 proteins in OVCAR5 cells with or without HORMAD1 (**e**) and WT and HORMAD1 KO MDAH2774 cells (**f**). α-tubulin and UHRF1 were used as loading controls for cytoplasm and nucleus, respectively. Histograms of nuclear and cytosol quantifications are shown on right. **g** Western blotting analyses of cytosolic and nuclear MCM8 and MCM9 proteins in MDAH2774 cells with or without HA-MCM9 HIM. *α*-tubulin and UHRF1 were used as loading controls for cytosolic and nuclear proteins, respectively. Histograms of nuclear and cytosol quantifications are shown on right. **h** Western blotting analyses of chromatin bound and nonchromatin bound MCM8 and MCM9 proteins in MDAH2774 cells with or without HA-MCM9 HIM. α-tubulin and UHRF1 were used as loading controls for nonchromatin and chromatin fractions, respectively. Mean ± SEM from three independent experiments are shown. **p < 0.01.

The above studies strongly suggest that HORMAD1 compromises mismatch repair by interacting with MCM8–MCM9 complex and disrupting its functions, but the mechanisms require further investigation. We first examined the expression levels of MCM8 and MCM9, but they remained the same after HORMAD1 expression in all the cells tested (Figs. 5a, b, S6A, B). Interestingly, the chromatin binding of MCM8 and MCM9 was significantly reduced in OVCAR5 cells after HORMAD1 expression and was significantly increased after HORMAD1 KO in MDAH2774, A549, and HCC38 cells (Figs. 5a, b, S6A, B). These results suggest that HORMAD1 compromises the chromatin-binding of MCM8–MCM9 complex.

The physiological function of HORMAD1 in meiosis suggests that HORMAD1 localizes in nucleus. Unexpectedly, SFB-tagged HORMAD1 was present not only in nucleus but also in cytosol in OVCAR5 cells (Fig. 5c). Significant amount of endogenous HORMAD1 was present in cytosol in MDAH2774, A549, and HCC38 cells as well (Figs. 5d, S6C, D). This indicates that HORMAD1 can shuttle between nucleus and cytosol in cancer cells. MCM8 and MCM9 are well known nuclear proteins. Interestingly, they were found in cytosol as well (Figs. 5e, f, S6E–F). Importantly, when HORMAD1 was expressed in OVCAR5 cells, the amount of MCM8 and MCM9 protein in cytosol was significantly increased and that in nucleus was decreased (Fig. 5e). Similarly, the amount of MCM8 an MCM9 protein in cytosol was significantly decreased and that in nucleus was increased after HORMAD1 KO in MDAH2774, A549, and HCC38 cells (Figs. 5f, S6E–F). Therefore, MCM8–MCM9 complex redistributes from nucleus to cytosol after HORMAD1 expression. On the contrary, cisplatin or UV radiation does not affect the cellular distribution of MCM8 or MCM9, no matter if HORMAD1 is present or not (Fig. S7A–D), suggesting that the cellular distribution of MCM8–MCM9 complex is unlikely regulated by DNA damage.

When the interaction between endogenous HORMAD1 and MCM8–MCM9 complex was abolished in MDAH2774 cells stably expressing MCM9 HIM, the nuclear localization and chromatin binding of MCM8–MCM9 complex were fully restored to the level seen in HORMAD1 KO MDAH2774 cells (Fig. 5g, h). Therefore, HORMAD1 cytosolic retention of MCM8–MCM9 complex is the mechanism how HORMAD1 compromises the function of MCM8–9 complex. Together with the finding that releasing endogenous MCM8–MCM9 complex from HORMAD1 binding fully rescued the MLH1 chromatin binding and DNA mismatch repair defects in MDAH2774 cells, this observation strongly suggests that cytosolic retention of MCM8–MCM9 complex is the mechanism how HORMAD1 compromises DNA mismatch repair in cancer cells.

### HORMAD1-expressing cancers have increased mutation load and genomic instability

**Fig. 6:**
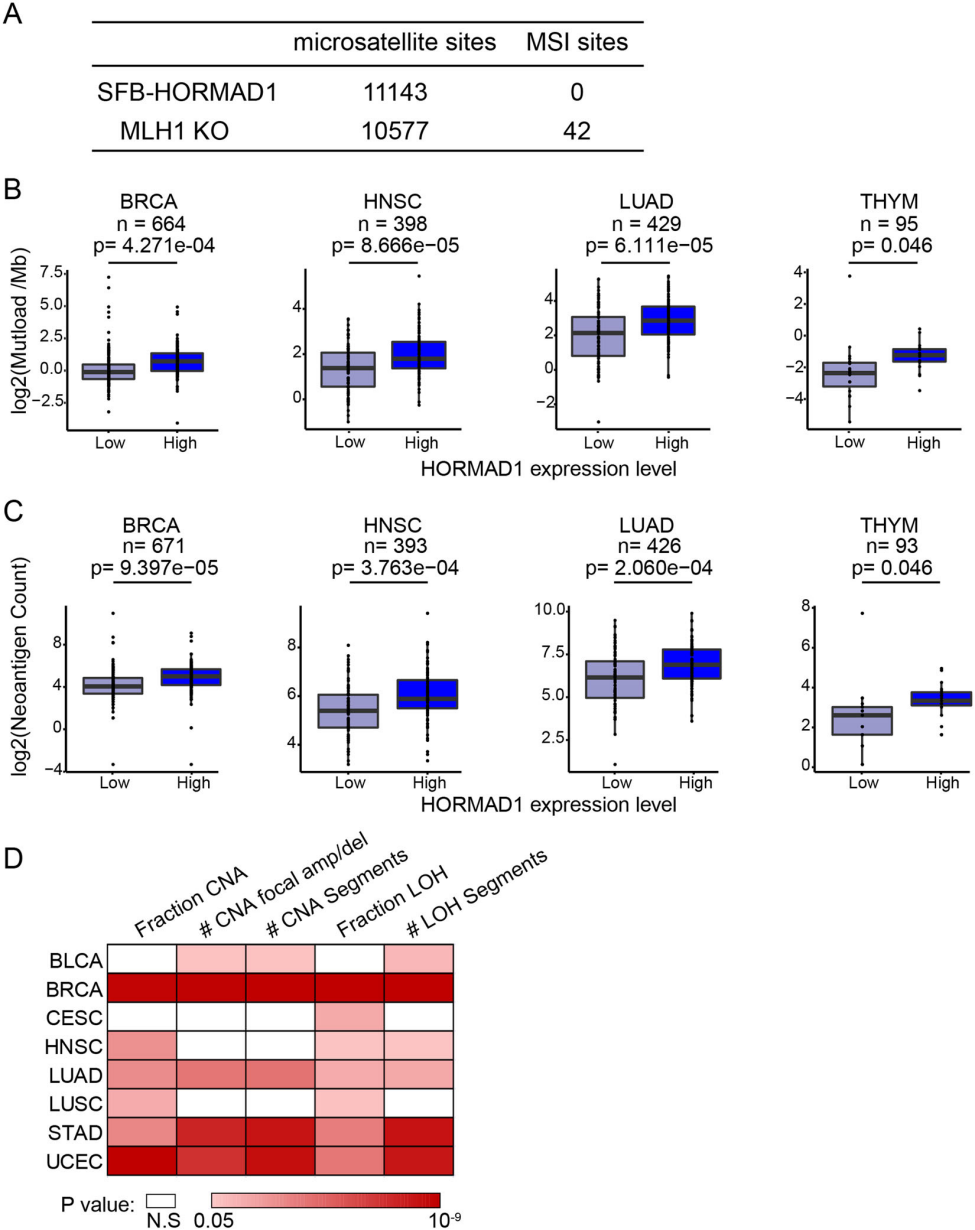
HORMAD1-expressing cancers have increased mutation load and genomic instability. **a** Genomic MSI sites identified by MSIsensor in OVCAR5 SFB-HORMAD1 and OVCAR5 MLH1 KO cells using OVCAR5 cells as control. **b**, **c**. Boxplots of log2-transformed non-silent mutation load per MB (**b**) and log2-transformed predicted neoantigen counts (**c**) in HORMAD1 expression high or low cancers. Two-tailed *t*-test was applied to calculate the significance between groups. To define HORMAD1 expression levels, the lowest 20% (HORMAD1-low) and the highest 20% (HORMAD1-high) samples are used for each type. **d** Heatmap shows the correlation of HORMAD1 expression with multiple genome instability features in TCGA cancers. Color intensity indicates the *p* value from the statistical tests. To calculate the significance between groups, rank-sum test was applied to fraction CNA and fraction LOH while *t*-test was applied to the others. Two-tailed test was utilized for all analyses. To define HORMAD1 expression levels, the lowest 20% (HORMAD1-low) and the highest 20% (HORMAD1-high) samples are used for each type.

Since DNA mismatch repair deficiency results in failure to identify and correct errors during DNA replication, it often leads to increased mutation load in cells, especially at sites with repetitive sequence known as microsatellites. This condition, commonly known as microsatellite instability (MSI), is another hallmark of DNA mismatch repair deficiency, which are often used in clinic to identify DNA mismatch repair deficient cancers. Given that HORMAD1 compromises DNA mismatch repair, we examined if HORMAD1 expression leads to MSI in cells. Genomic DNA from OVCAR5 cells with or without stable HORMAD1 expression and MLH1 KO OVCAR5 cells were extracted and subjected to whole exome sequencing. MSIsensor software was used to scan genomic microsatellite sites in these cells and to identify MSI sites^23^. Forty-two MSI sites were identified in MLH1 KO OVCAR5 cells, suggesting that these cells have increased level of MSI (Fig. 6a). Surprisingly, no MSI sites were identified in OVCAR5 cells with HORMAD1 expression (Fig. 6a), which indicates that these cells have very low MSI levels. This result suggests aberrant HORMAD1 expression does not increase mutation load despite compromising DNA mismatch repair in cancer cells.

To further interrogate the cellular studies, we investigated the mutation load status in human cancer samples with or without HORMAD1 expression in TCGA database. Interestingly, HORMAD1 expression was associated with increased tumor mutation load in several cancers (Fig. 6b). Consistent with the idea that elevated mutation load leads to the generation of neoantigens that binds MHC proteins and induces antitumor adaptive immunity, HORMAD1 expression was associated with increased neoantigen counts in these cancers (Fig. 6c). These observations suggest that, in contrast to our cellular studies, HORMAD1 expression increases mutation load in some cancers. Unlike MLH1 KO, HORMAD1 expression does not completely abolished MLH1 chromatin binding. It is possible that much longer time and more rounds of DNA replication are required before compromised DNA mismatch repair can caused increased mutation load after HORMAD1 expression. Therefore, the drastic difference in the duration of HORMAD1 expression (weeks vs. years) might underline the mutation load difference between cells and cancers. Additional factors might trigger the mutation load increase in cancers after HORMAD1 expression as well (see “Discussion” section).

Previous studies have shown that triple-negative breast cancers with aberrant HORMAD1 expression is associated with genomic instability^12^. Since HORMAD1 compromises DNA mismatch repair, an important component in the maintenance of genomic stability, it is possible that HORMAD1 expression is commonly associated with genomic instability in cancers. To test this possibility, we included all group I cancers that have distinct HORMAD1-positive populations (Fig. 1b) and examined if HORMAD1 expression was associated with features of genomic instability, including copy number alternation burdens (number of segments altered, number of local amplification/deletion, and fraction of genome altered) and loss of heterozygosity (LOH) burdens (number of segments with LOH and fraction of genome with LOH)^24^. Significant positive correlations between HORMAD1 expression and various genomic instability features were identified in 8 out of 12 group I cancers, suggesting that HORMAD1 expression is indeed associated with genomic instability in many cancers (Fig. 6d). It will be interesting to investigate in future if compromised DNA mismatch repair directly contributes to genomic instability in these cancers.

## Discussion

**Fig. 7:**
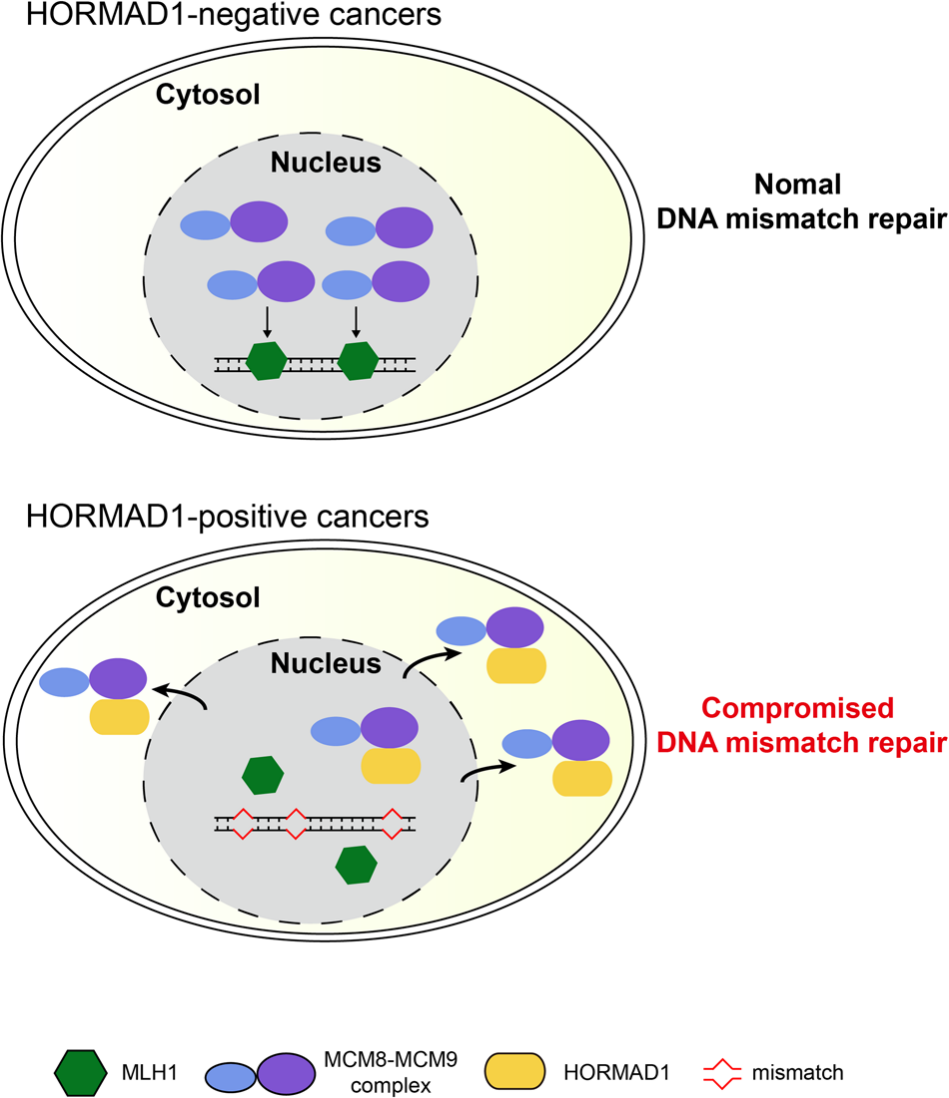
Working model: HORMAD1 compromises DNA mismatch repair. In HORMAD1-negative cancer cells, nuclear MCM8–MCM9 complex promotes MLH1 chromatin loading and DNA mismatch repair. In HORMAD1-positive cancer cells, HORMAD1 dirupts nuclear localization of MCM8–MCM9, reduces MLH1 chromatin loading, and compromises DNA mismatch repair.

In this study, we have shown that meiosis-specific protein HORMAD1 is widely expressed in many cancers. Aberrantly expressed HORMAD1 binds to and prevents efficient nuclear localization of MCM8-MCM9 complex, which leads to reduced MLH1 loading and compromised DNA mismatch repair (Fig. 7). This is the first time that the function and mechanism of HORMAD1 in cancer cells are discovered. Given the role of HORMAD1 in disrupting normal cellular functions, our study suggests that HORMAD1 might not be passively expressed by global hypomethylation in cancers, but might be selected to facilitate cancer development. This possibility is consistent with our finding that HORMAD1 is widely expressed in many cancers.

DNA mismatch repair machinery has been identified for a long time. In some cancers, genes encoding core components of the DNA mismatch repair machinery, such as MSH2 and MLH1, are frequently mutated or silenced^25^. Compared with the core components, the regulators of DNA mismatch repair machinery are not well characterized. MCM8–MCM9 complex regulates MLH1 chromatin loading and DNA mismatch repair^20^, but their status in cancers are not examined. A recent study has identified ARID1A as a critical regulator of DNA mismatch repair that interacts with MSH2 and promote MSH2’s chromatin loading^26^. Consistently, ARID1A is frequently mutated in many cancers^26^. Here we have identified HORMAD1 as a unique negative regulator of DNA mismatch repair machinery that is specifically expressed in cancers but not in normal somatic cells. For the first time, our study reveals that an aberrant expressed protein can disrupt DNA mismatch repair without affecting the expression level of core components or regulators of the DNA mismatch repair machinery. This novel mechanism suggests there are more ways to compromise DNA mismatch repair in cancers.

Given the reduced MLH1 chromatin binding and the decreased sensitivity to 6-TG in *HPRT* assay, it is clear that HORMAD1 compromises DNA mismatch repair. However, HORMAD1 expression does not increase mutation load as MLH1 KO does in culture cells. This unexpected finding is likely due to the fact that HORMAD1 does not completely abolish the chromatin loading of MLH1. It is possible that after HORMAD1 expression, the remaining MLH1 on chromatin is sufficient for DNA mismatch repair under normal conditions but is not enough for conditions with increased DNA mismatches, such as after 6-TG treatment. In addition, the time of culture might not be enough to cause significant mutation load increase when DNA mismatch repair is compromised but not completely abolished.

Interestingly, our analyses reveal that HORMAD1 expression is associated with increased mutation load and increased neoantigen counts in several cancers, which indicates that additional factors trigger the generation of mutations in these cancers. Since cancer development takes several years, which is much longer than cells in culture, the chances of generating mutations are much higher after much more rounds of DNA replication with compromised DNA mismatch repair. A recent study has also found that replication stress is important for triggering increased mutation load and MSI in cancers with DNA mismatch repair deficiency^27^. Since replication stress is a hallmark of cancers^28,29^, it is likely that cancers has a unique environment so that replication stress couples with HORMAD1-mediated compromised DNA mismatch repair to increase mutation load in HORMAD1-positive cancers.

Recent studies have shown that DNA mismatch repair deficient cancers have exceptional response to anti-PD-1 antibodies^30,31^. In addition, high mutation load and MSI can also predict response to immune checkpoint blockade therapy^32–35^. Since HORMAD1 compromises DNA mismatch repair and HORMAD1 is associated with increased mutation load and increased neoantigen counts in several cancers, it is possible that HORMAD1-expressing cancers can respond to immune checkpoint blockade therapy. Since HORMAD1 expression does not increase mutation load in cultured cells in vitro, it is difficult to test this idea using mouse tumor cells in immune-competent mouse models. However, it might be feasible to use HORMAD1-expressing human cancer samples to establish PDX models in humanized immune-competent mice and test this idea in future. Nevertheless, our findings indicate that the compromised DNA mismatch repair has the potential to be exploited for targeted therapy of HORMAD1-expressing cancers. Since HORMAD1 is widely expressed in many cancers, such therapy will potentially benefit a large group of patients with HORMAD1-expressing cancers.

## Materials and methods

### TCGA data analysis

The mRNA expression data was downloaded from TCGA Data Portal and log2-transformed. Genomic instability features of the corresponding TCGA samples was obtained from a previous study^24^. Predicted neoantigen counts from mutations was obtained from another study^36^. Statistical analyses were performed and plotted in R (version version 3.6.1), using packages “survival” and “ggplot2”.

TCGA cancers were divided into three sub-groups based on their HORMAD1 expression distribution. First, log2-transformed HORMAD1 expression was plotted for their distribution in all TCGA cancers with sample size greater than 50. Then the following heuristic criteria was applied: for distribution plot with bimodal shape, if the higher peak has a log2-transformed expression value greater than 4, then the cancer is defined as group I. For distribution plot without a significant bimodal shape, if the distribution is right-skewed with a distinct percentage of samples with high-expression (percentage > 4.5%), the cancer is defined as group II. If the distribution is approximately normal without any highly expressed outliers, the cancer is defined as group III. Based on the above criteria, there are 12 cancers in group I (not including TGCT in which most cancers express HORMAD1), 6 cancers in group II, and 6 cancers in group III.

To detect the correlation between HORMAD1 expression and features including genomic instability features, mutation burden and neoantigen count in TCGA samples, HORMAD1-high (top 20%) and HORMAD1-low (low 20%) was defined as “high” and “low” groups. The correlation was analyzed by statistical tests specified in figure legends.

### Cell culture and transfection

293T and U2OS cells were from ATCC. All ovarian cancer cells were gifts from Xiaochun Yu (City of Hope). A549 and HCC38 cells were gifts from Yongchao Zhao (Zhejiang University). All cells except HCC38 were maintained in DMEM supplemented with 10% fetal bovine serum and 1% penicillin and streptomycin. HCC38 cells were cultured in RPMI 1640 supplemented with 10% fetal bovine serum and 1% penicillin and streptomycin. Cell transfection was carried out using Lipofectamine 3000 (Invitrogen) transfection reagent according to the manufacturer’s instructions.

### DNA constructs

MCM8 and MCM9 cDNA were gifts from Masato Kanemaki (National Institute of Genetics, Japan). HORMAD1 cDNA was amplified by PCR using cDNA from MDAH2774 cells. Overlapping PCR was performed according to standard procedures to obtain deletion mutants. All constructs were confirmed by DNA sequencing. The details of the deletion mutants are: HORMAD1ΔC (Δ237–394 amino acids), MCM8–D1 (Δ1–355 amino acids), MCM8–D2 (Δ356–680 amino acids), MCM8–D3 (Δ681–840 amino acids), MCM9–D1 (Δ1–260 amino acids), MCM9–D2 (Δ261–535 amino acids), MCM9–D3 (Δ536–840 amino acids), MCM9–D4 (Δ841–1143 amino acids), MCM9–D3.1 (Δ536–595 amino acids), MCM9–D3.2 (Δ596–645 amino acids), MCM9–D3.3 (Δ646–702 amino acids), MCM9-D3.4 (referred to as ΔHIM, Δ703–766 amino acids), MCM9–D3.5 (Δ767–840 amino acids).

### Generation of cells stably expressing HORMAD1

293T and OVCAR5 were transfected with S-FLAG-streptavidin binding protein (SFB) triple-tagged HORMAD1 expression construct and cultured in medium containing 2 μg/ml puromycin for 7 days. Individual clones resistant to puromycin were then picked and expanded. SFB-HORMAD1 expression was confirmed by western blotting.

### Generation of HORMAD1 KO and MLH1 KO Cells

HORMAD1 KO and MLH1 KO cells were generated using CRISPR/Cas9 technology. The guide RNA sequences for HORMAD1 are 5′-TCTTCACTAACACCAAAGAC-3′ (KO1) and 5′-TCCTGTATCACGTATTTGAG-3′ (KO2). The guide RNA sequence for MLH1 is 5′-TTTTTTACAACATAGCCACG-3′. Guide RNAs were cloned into the PX459 V2.0 plasmids (gifts from Feng Zhang, Addgene 62988) according to standard protocols. Cells were transfected with PX459 V2.0 guide RNA constructs and cultured in medium containing 2 μg/ml puromycin for 48 h. Individual clones resistant to puromycin were then picked and expanded. KO cells were confirmed by western blotting.

### Tandem affinity purification

293T cells stably expressing SFB-tagged HORMAD1 from 50 10 cm^2^ culture dishes were collected and lysed with NETN300 buffer (50 mM Tris-HCl pH8.0, 300 mM NaCl, 0.5 mM EDTA, 0.5% Nonidet P-40) for 20 min on ice. The supernatants were diluted with the same volume of ddH_2_O and incubated with streptavidin-conjugated beads at 4 °C for 2 h. The beads were washed three times with NETN100 buffer (50 mM Tris-HCl pH 8.0, 100 mM NaCl, 0.5 mM EDTA, 0.5% Nonidet P-40) and eluted with saturating biotin (Sigma) in NETN100 buffer for 30 min at 4 °C. The eluants were incubated with S-protein agarose beads (Millipore) for 2 h at 4 °C. The beads were washed three times with NETN100 buffer. Proteins bound to S-beads were eluted by SDS loading buffer and subjected to sodium dodecyl sulfate polyacrylamide gel electrophoresis (SDS-PAGE) briefly. The entire protein band (less than 1 cm) was excised and analyzed by mass spectrometry.

### Mass spectrometry analysis

Gel bands were cut into 1 mm^3^ pieces and were subjected to in-gel trypsin digestion overnight. The peptides were extracted with acetonitrile and vacuum dried. Samples were loaded onto Proxeon EASY-nLC II liquid chromatography pump (Thermo Fisher) after reconstituted in HPLC solvent A (2.5% acetonitrile, 0.1% formic acid). By increasing the concentration of solvent B (97.5% acetonitrile, 0.1% formic acid), samples were eluted with a gradient of acetonitrile (6–30%) within 30 min. The eluates were directly subjected to Orbitrap Elite MS (Thermo Fisher). To produce a tandem mass spectrum of specific fragment ions for each peptide, the peptides were detected, isolated, and fragmented. The MS/MS spectra were analyzed by matching protein databases with the acquired fragmentation patterns using SEQUEST (ver. 28, Thermo Fisher). Enzyme specificity was set to partially tryptic with two missed cleavages. Carboxyamidomethyl for cysteine and oxidation for methionine residues were set as static modifications and variable modification respectively. According to the target-bait method, the identified peptides were filtered with false discovery rate (FDR) < 1%. A complete list of peptides identified by mass spectrometry was shown in Table S1.

### Antibodies

Anti-MCM9 antibody was generated by immunizing rabbits with GST-MCM9 (residues 936–1135) in HuaBio. The following antibodies were purchased: anti-MCM8 (Proteintech, 1645-1-AP), anti-HORMAD1 (Proteintech, 13917-1-AP), anti-MSH2 (Proteintech, 15520-1-AP), anti-MLH1 (Santa Cruz, sc-271978), anti-UHRF1 (Santa Cruz, sc-373750), anti-α-tubulin (Genscript, A01410-100), anti-FLAG (Sigma, F1804), anti-HA (Sangong, D199961), anti-*γ*H2AX (Abcam, ab81299), and anti-RAD51 (Santa Cruz, sc-8349).

### Co-immunoprecipitation and western blotting

Cells were harvested and lysed with NETN300 buffer for 10 min on ice. The supernatants were diluted with the same volume of ddH_2_O and incubated with streptavidin-conjugated beads or 2 μg of indicated antibodies and 40 μl protein A sepharose beads for 2 h at 4 °C. The beads were washed three times with NETN100 buffer. The bound protein was eluted using SDS loading buffer and resolved on SDS-PAGE. All Western blotting experiments were performed according to standard procedures.

### Homologous recombination assay

Cells with the indicated genotypes were plated onto 6-well plates and transfected with DR-GFP plasmids (gifts from Maria Jasin, Memorial Sloan Kettering Cancer Center). Plasmids expressing GFP were separately transfected into cells to evaluate the transfection efficiency at the same time. After transfection for 24 h, cells were infected with I-SceI expressing adenovirus for 24 h and harvested for flow cytometry to analysis the GFP positive cells. HR efficiency were normalized by transfection efficiency and results were normalized to wild-type cells.

### PARPi sensitivity assay

5 × 10^2^ cells were seeded onto 6-well plates and treated with olaparib at indicated concentrations. Medium was changed every day. After 7 days exposure to olaparib, cells were fixed in 4% paraformaldehyde and stained with 0.5% crystal violet. To quantify colonies, cells stained with crystal violet were dissolved in a 10% acetic acid solution and the absorbance at OD595 was measured.

### Immunofluorescence staining

Cells cultured on coverslips were treated with 10 μM cisplatin for 6 h. Cells were fixed with 4% paraformaldehyde for 10 min and permeabilized with 0.5% Triton X-100 for 5 min. After washing with PBS, cells were incubated with indicated primary antibodies for 1 h at room temperature. Cells were subsequently incubated with Alexa Fluor 488 or 594 labeled secondary antibody (Jackson ImmunoResearch) for 30 min at room temperature. Coverslips were then stained with Hoechst 33342, mounted with anti-fade solution, and visualized using a fluorescence microscope (Eclipse Ti2, Nikon). Colocalization analysis was performed using confocal microscopy (TCS SP8, Leica).

### *HPRT* gene mutation (6-TG sensitivity) assay

2 × 10^3^ cells were seeded onto 24-well plates. Twenty-four hours later, cells were treated with 6-TG at indicated concentrations for 7 days. Medium was changed every day. Cell viability was measured by CCK8 reagent (DOJINDO) following the manufacturer’s instructions.

### Separation of chromatin-bound and nonchromatin-bound proteins

Cell pellets were lysed with NETN100 buffer on ice for 10 min. After centrifugation, supernatants were used as nonchromatin fractions. Pellets were washed three times with ice-cold PBS and dissolved in 0.2 M HCl. After centrifugation, supernatants were neutralized with Tris-HCl (1 M at pH 8.0) and used as chromatin fractions.

### Separation of cytosolic and nuclear proteins

Cells were lysed with cytosolic extraction buffer (10 mM Tris–HCl, pH 8.0, 10 mM KCl, 1.5 mM MgCl_2_, 1 mM EDTA) and incubated on ice for 10 min. Four microliter of of 10% nonidet P-40 were then added, and the mixture was vortexed and incubated on ice for 2 min. After centrifugation, supernatants were collected and used as cytosolic extract. Pellets were washed three times with ice-cold PBS and dissolved in nuclear extraction buffer (10 mM Tris–HCl, pH 8.0, 400 mM NaCl, 1.5 mM MgCl_2_, 1 mM EDTA, 5% glycerol) for 10 min on ice. After centrifugation, supernatants were collected and used as nuclear extract.

### Whole exome sequencing and bioinformatics analysis

OVCAR5 and its related cell lines were passaged continuously under standard growth conditions. Cells that were passaged for 4 weeks were collected and underwent 100× paired-end 150 bp whole-exome sequencing at Novogene. Raw sequencing data were aligned to GRCh38 reference genome by Burrows–Wheeler Alginer mem algorithm (BWA-mem v 0.7.12-r1039) with default parameters^37^. Reads were sorted by samtools (version 1.1) and duplicated reads were removed by Picard (v 2.0.1) (“Picard Toolkit.” 2019, Broad Institute, GitHub Repository)^38^. To evaluate genomic microsatellite status, MSIsensor (Version 0.6) was applied to the aligned bam files with default parameters using parental OVCAR5 cells as control^23^. Whole-exome sequencing data has been deposited in NCBI SRA database (PRJNA632438).

### Statistics

All data in bar and line graphs are presented as mean ± SEM of at least three independent experiments. All data were analyzed by the two-tailed unrepaired student’s *t*-test or two-way ANOVA using GraphPad prism version 7.0. *p < 0.05, **p < 0.01, ***p < 0.001, and ns denotes not significant.

## Supplementary information

Supplementary figure legends

Figure S1

Figure S2

Figure S3

Figure S4

Figure S5

Figure S6

Figure S7

Table S1
