# Supplementary material for: Aberrantly expressed HORMAD1 disrupts nuclear localization of MCM8–MCM9 complex and compromises DNA mismatch repair in cancer cells
Source: Cell Death Dis. 2020 Jul 9;11(7):519. doi: 10.1038/s41419-020-2736-1 (PMC7347845; doi:10.1038/s41419-020-2736-1)
Supplement: Supplementary file 1 — Supplementary figure legends [file 41419_2020_2736_MOESM1_ESM.docx]

**Supplementary Figure legends**

Figure S1. HORMAD1 is widely expressed in cancers.

Log2-transformed HORMAD1 expression distribution in TCGA cancers with sample size greater than 50. Red line represents for the cutoff between HORMAD1-positive (expressed) and HOMRAD1-negative (silent) samples and the HORMAD1-positive area is filled blue.

Figure S2. HORMAD1 interacts with MCM8-MCM9 complex.

**A-B.** A schematic representation of WT and deletion mutants of MCM8 (**A**) and MCM9 (**B**). **C.** Co-IP analyses of the interaction between SFB-MCM8 and HA-MCM9 deletion mutants in 293T cells. **D.** Co-IP analyses of the interaction between SFB-MCM9 and HA-MCM8 deletion mutants in 293T cells. **E.** Co-IP analyses of the interaction between SFB-HORMAD1 and HA-MCM8 deletion mutants in 293T cells. **F.** Co-IP analyses of the interaction between SFB-HORMAD1 and HA-MCM9 deletion mutants in 293T cells. **G.** A schematic representation of MCM9 D3 deletion mutants. **H.** Co-IP analyses of the interaction between SFB-HORMAD1 and HA-MCM9 D3 deletion mutants in 293T.

Figure S3. HORMAD1 promotes homologous recombination repair and PARPi resistance independent of its interaction with MCM8-MCM9 complex.

**A.** Schematic representation of DR-GFP reporter-based HR assay. **B.** Western blotting analyses of HORMAD1 expression in OVCAR5 cells with or without HORMAD1 expression. α-tubulin was used as loading control. **C.** Summary of HR efficiency in OVCAR5 cells with or without HORMAD1 expression. **D.** Cell viability of OVCAR5 cells with or without HORMAD1 expression after treatment with indicated doses of olaparib. **E.** Western blotting analyses of HORMAD1 expression in HORMAD1 WT and KO MDAH2774 cells. α-tubulin was used as loading control. **F.** Summary of HR efficiency in HORMAD1 WT and KO MDAH2774 cells. **G.** Cell viability of HORMAD1 WT and KO MDAH2774 cells after treatment with indicated doses of olaparib. **H.** Western blotting analyses of HORMAD1 protein in WT and HORMAD1 KO A549 cells. α-tubulin was used as loading control. **I.** Summary of HR efficiency in WT and HORMAD1 KO A549 cells. **J**. Cell viability analyses of in WT and HORMAD1 KO A549 cells after treatment with indicated doses of olaparib. **K.** Western blotting analyses of HORMAD1 protein in WT and HORMAD1 KO HCC38 cells. α-tubulin was used as loading control. **L.** Summary of HR efficiency in WT and HORMAD1 KO HCC38 cells. **M**. Cell viability analyses of in WT and HORMAD1 KO HCC38 cells after treatment with indicated doses of olaparib. **N.** Summary of HR efficiency in MDAH2774 cells with or without HA-MCM9 HIM. **O.** Cell viability analyses of in MDAH2774 cells with or without HA-MCM9 HIM after treatment with indicated doses of olaparib. Mean ± SEM from three independent experiments are shown. *, p＜0.05; **, p＜0.01; ns, not significant.

Figure S4. HORMAD1 expression has no effect on the recruitment of RAD51 to DNA break sites.

**A-B.** Immunofluorescence staining analyses of RAD51 foci in OVCAR5 cells with or without HORMAD1 expression (**A**) and in HORMAD1 WT and KO MDAH2774 cells (**B**) after 6 hours of 10 μM cisplatin treatment. **C-D.** Immunofluorescence staining analyses of RAD51 foci in WT and HORMAD1 KO A549 (**C**) and HCC38 (**D**) cells after 6 hours of 10μM cisplatin treatment. γH2AX represents DNA damage site. Hoechst 33342 stains nucleus. Quantifications of RAD51 foci are shown below each panel. Scale bar, 10 μm. Mean ± SEM from three independent experiments are shown. ns, not significant.

Figure S5. HORMAD1 expression compromises DNA mismatch repair.

**A-B.** Cell viability of in WT and HORMAD1 KO A549 (**A**) and HCC38 (**B**) cells after treatment with indicated doses of 6-TG. **C-D.** Western blotting analyses of total, chromatin-bound, and non-chromatin-bound MSH2 and MLH1 proteins in WT and HORMAD1 KO A549 (**C**) and HCC38 (**D**) cells. α-tubulin and UHRF1 were used as loading controls for non-chromatin and chromatin fractions, respectively. Mean ± SEM from three independent experiments are shown. **, p＜0.01.

Figure S6. HORMAD1 compromises nuclear localization of MCM8-MCM9 complex.

**A-B.** Western blotting analyses of total, chromatin-bound, and non-chromatin-bound MCM8 and MCM9 proteins in WT and HORMAD1 KO A549 (**A**) and HCC38 (**B**) cells. α-tubulin and UHRF1 were used as loading controls for non-chromatin and chromatin fractions, respectively. **C-D.** Western blotting analyses of cytosolic and nuclear HORMAD1 protein in WT and HORMAD1 KO A549 (**C**) and HCC38 (**D**) cells. α-tubulin and UHRF1 were used as loading controls for cytosolic and nuclear proteins, respectively. **E-F.** Western blotting analyses of cytosolic and nuclear MCM8 and MCM9 proteins in WT and HORMAD1 KO A549 (**E**) and HCC38 (**F**) cells. α-tubulin and UHRF1 were used as loading controls for cytosolic and nuclear proteins, respectively.

Figure S7. Cellular distribution of MCM8-MCM9 complex is not regulated by DNA damage.

**A-B.** Western blotting analyses of cytosolic and nuclear MCM8 and MCM9 proteins in OVCAR5 cells with or without HORMAD1 (**A**) and WT and HORMAD1 KO MDAH2774 cells (**B**) after 6 hours of 20 μM cisplatin treatment. α-tubulin and UHRF1 were used as loading controls for cytoplasm and nucleus, respectively. Histograms of nuclear and cytosol quantifications are shown on right. **C-D.** Western blotting analyses of cytosolic and nuclear MCM8 and MCM9 proteins in OVCAR5 cells with or without HORMAD1 (**C**) and WT and HORMAD1 KO MDAH2774 cells (**D**) 4 hours after 40 J/m^2^ UV treatment. α-tubulin and UHRF1 were used as loading controls for cytoplasm and nucleus, respectively. Histograms of nuclear and cytosol quantifications are shown on right. Mean ± SEM from three independent experiments are shown. **, p＜0.01.
