# Supplementary figures and images for: Aberrantly expressed HORMAD1 disrupts nuclear localization of MCM8–MCM9 complex and compromises DNA mismatch repair in cancer cells
Source: Cell Death Dis. 2020 Jul 9;11(7):519. doi: 10.1038/s41419-020-2736-1 (PMC7347845; doi:10.1038/s41419-020-2736-1)

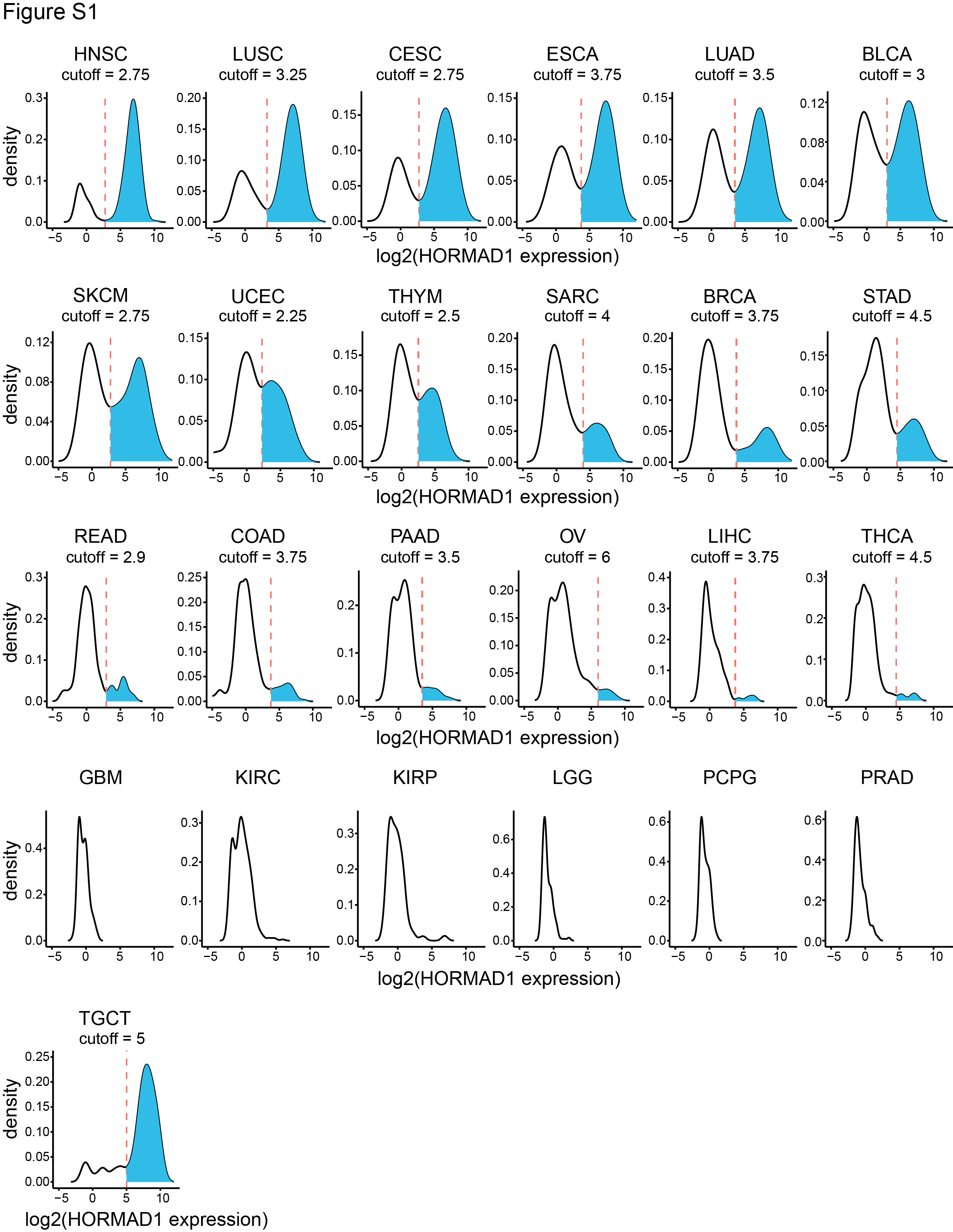

Supplement: Supplementary file 2 — Figure S1 [file 41419_2020_2736_MOESM2_ESM.png]

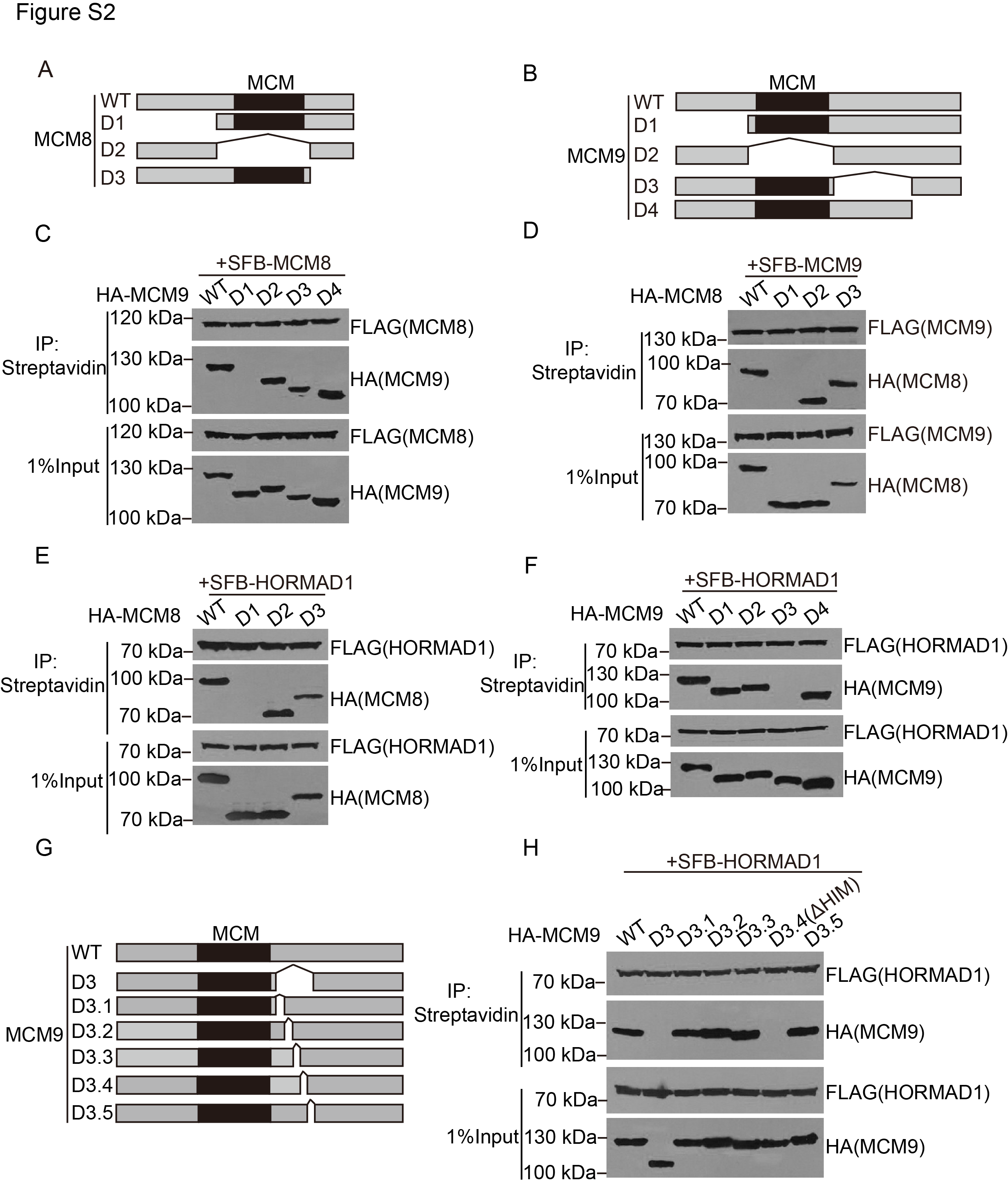

Supplement: Supplementary file 3 — Figure S2 [file 41419_2020_2736_MOESM3_ESM.png]

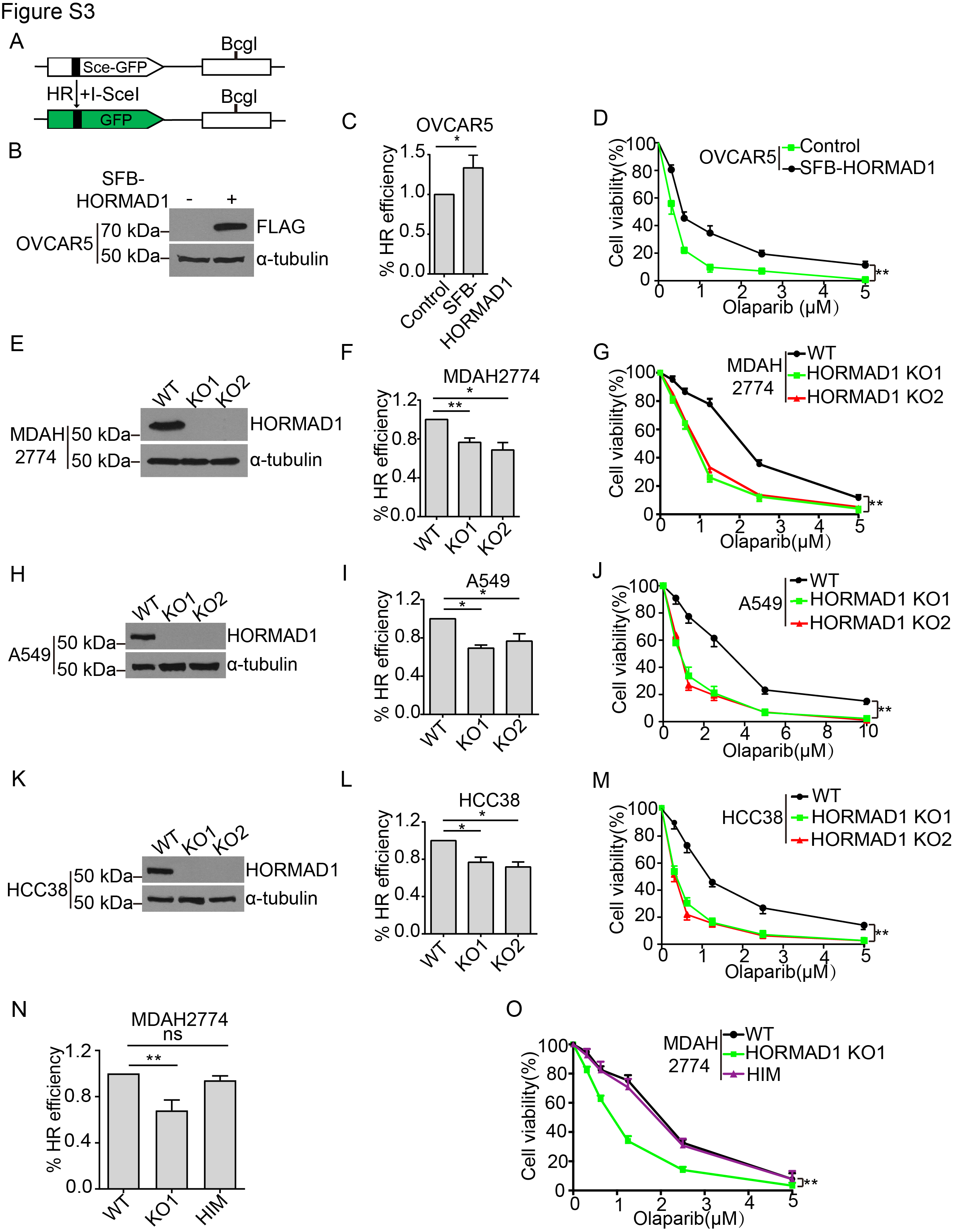

Supplement: Supplementary file 4 — Figure S3 [file 41419_2020_2736_MOESM4_ESM.png]

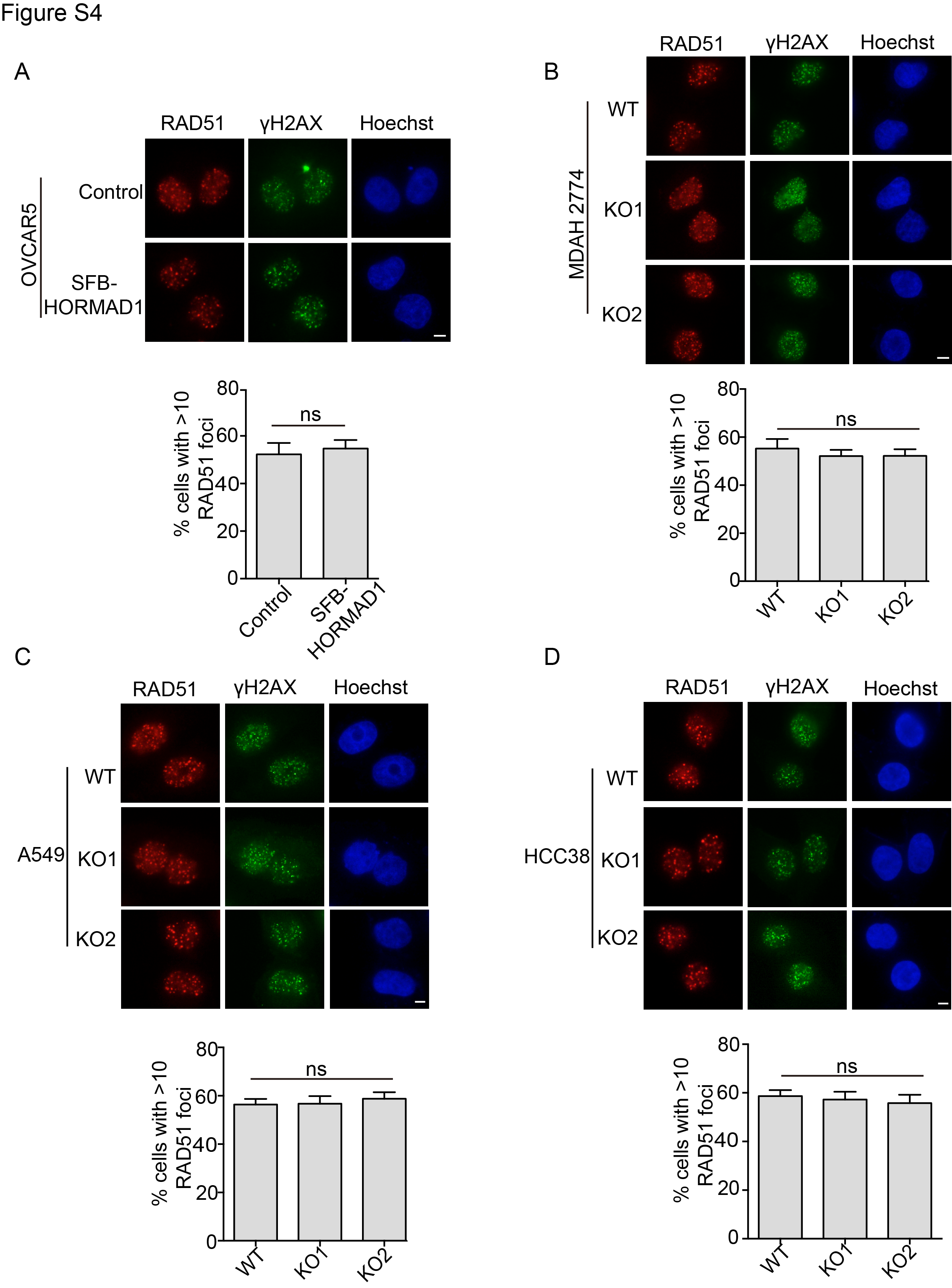

Supplement: Supplementary file 5 — Figure S4 [file 41419_2020_2736_MOESM5_ESM.png]

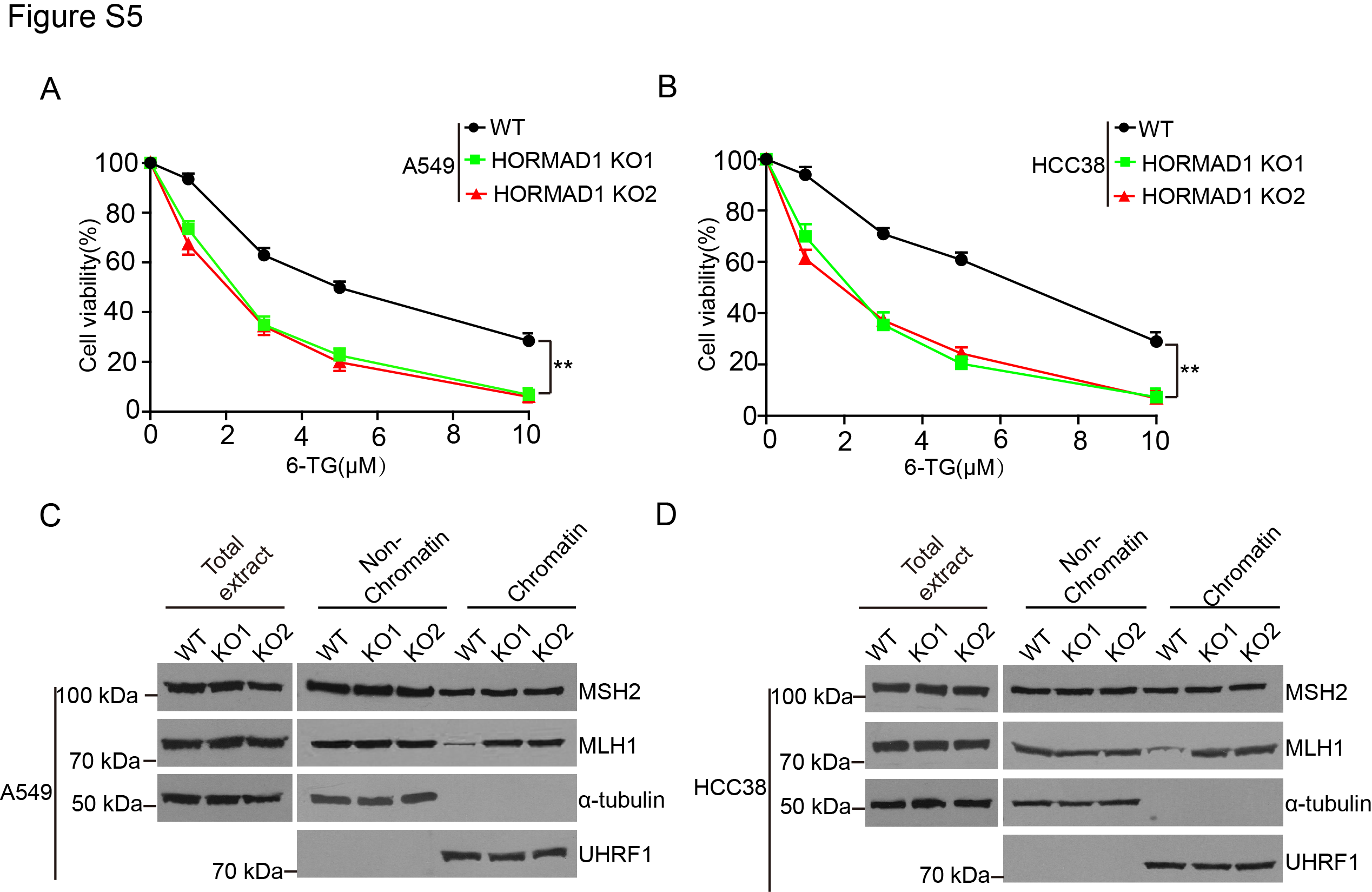

Supplement: Supplementary file 6 — Figure S5 [file 41419_2020_2736_MOESM6_ESM.png]

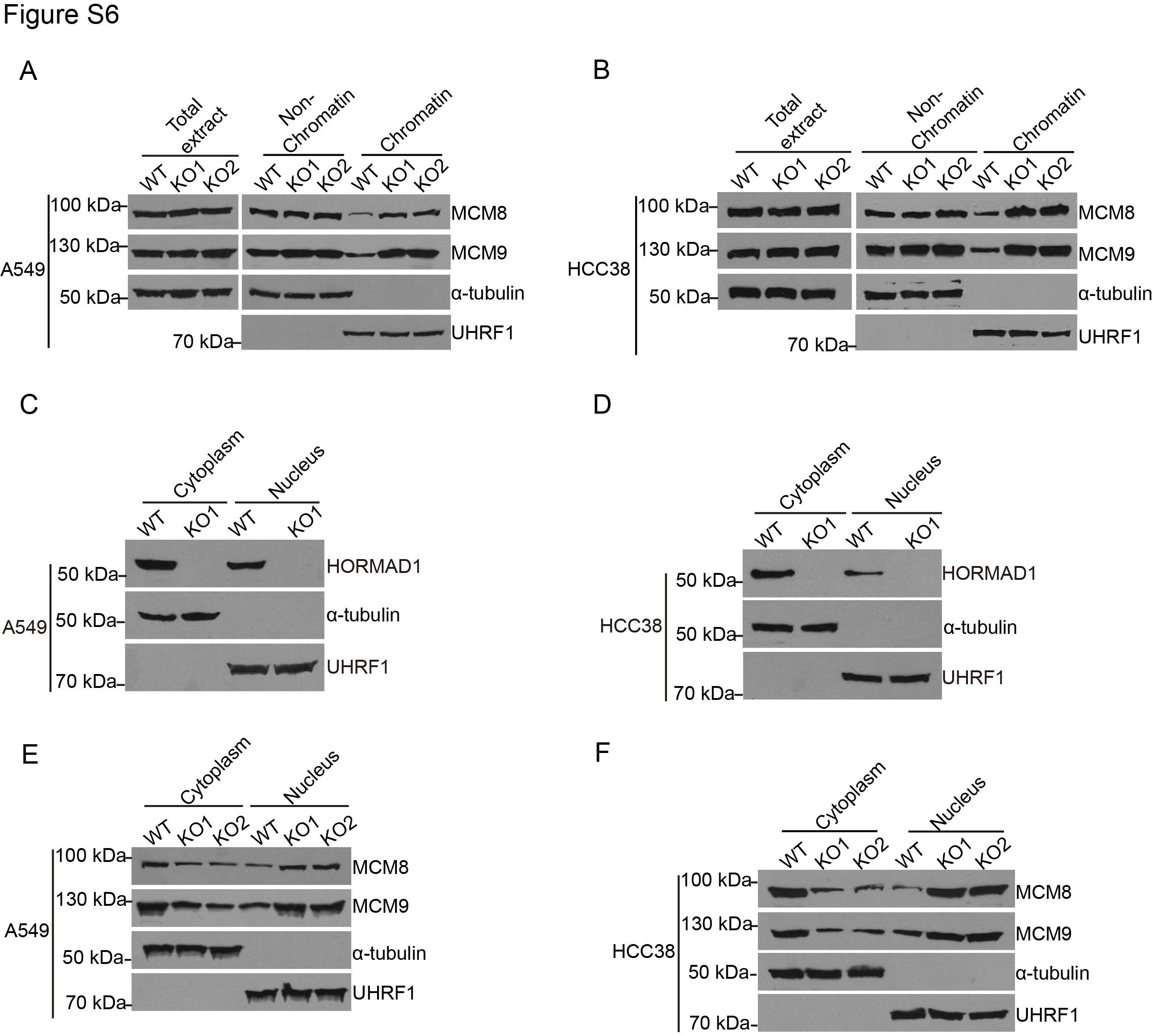

Supplement: Supplementary file 7 — Figure S6 [file 41419_2020_2736_MOESM7_ESM.png]

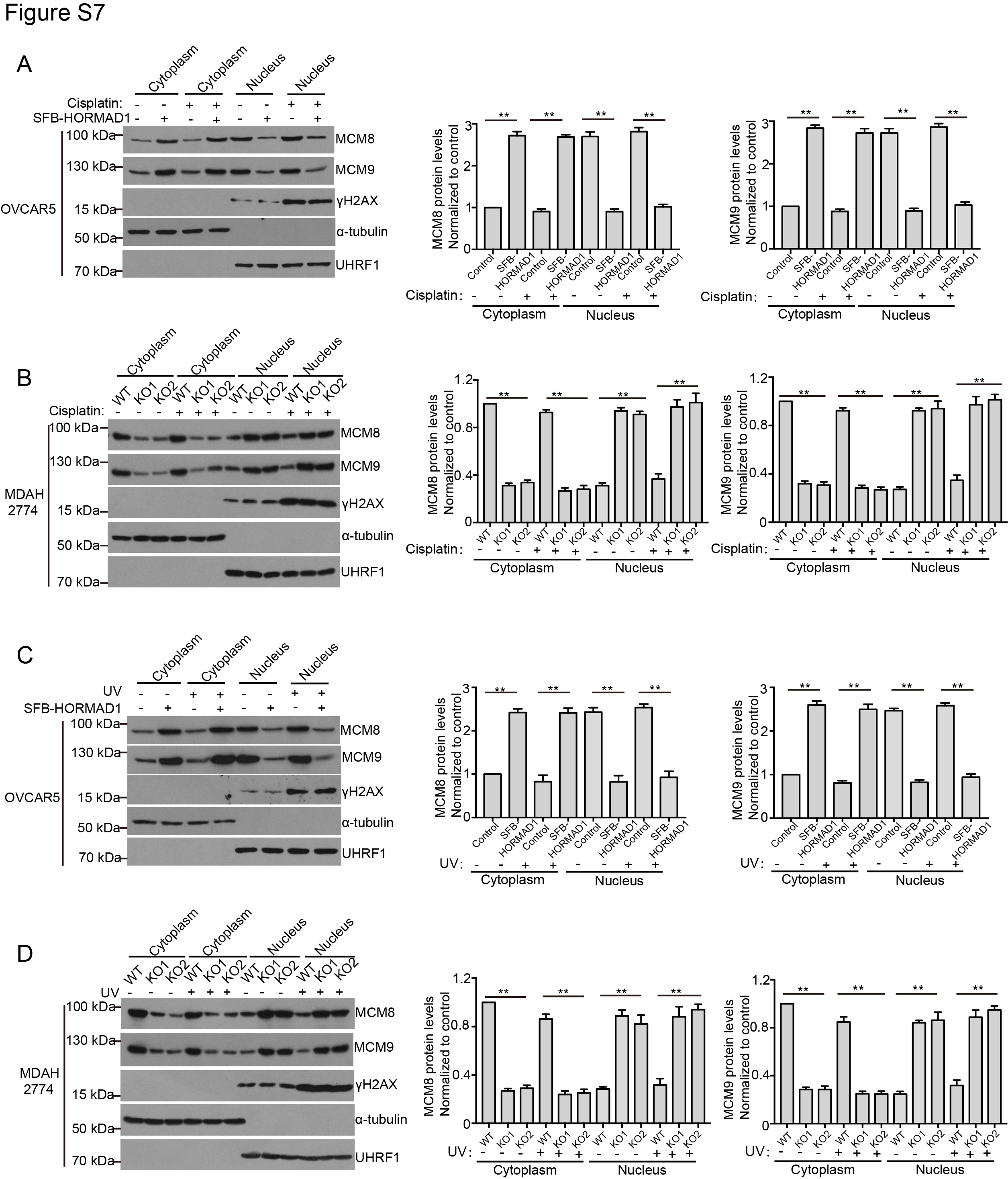

Supplement: Supplementary file 8 — Figure S7 [file 41419_2020_2736_MOESM8_ESM.png]
